# Maintenance of sinus rhythm after electrical cardioversion to identify patients with persistent atrial fibrillation who respond favorably to pulmonary vein isolation: the pre-pacific study

**DOI:** 10.3389/fcvm.2024.1416975

**Published:** 2024-10-11

**Authors:** Luca Rosario Limite, Guillaume Laborie, F. Daniel Ramirez, Jean-Paul Albenque, Stephane Combes, Philippe Lagrange, Ziad Khoueiry, Agustín Bortone

**Affiliations:** ^1^ Service de Cardiologie, Hôpital Privé Les Franciscaines, ELSAN, Nîmes, France; ^2^ Service de Cardiologie, Clinique Saint Pierre, ELSAN, Perpignan, France; ^3^ Division of Cardiology, University of Ottawa Heart Institute, Ottawa, ON, Canada; ^4^ School of Epidemiology and Public Health, University of Ottawa, Ottawa, ON, Canada; ^5^ Département de Rythmologie, Clinique Pasteur, Toulouse, France

**Keywords:** persistent atrial fibrillation, electrical cardioversion, sinus rhythm, atrial remodeling, pulmonary vein isolation

## Abstract

**Background:**

Pulmonary vein isolation (PVI) is successful in approximately 50% of patients with persistent atrial fibrillation (PsAF) at one year. Identifying pre-procedurally the patients who respond favorably to a PVI alone strategy could improve their management. The present study aims to assess the predictive value of clinical response to pre-ablation electrical cardioversion (ECV) to identify the responders to PVI.

**Methods:**

Consecutive patients undergoing catheter ablation for PsAF were retrospectively classified, as “ECV successful” vs. “ECV failure”, according to the rhythm of presentation after an ECV performed ≥4 weeks. Clinical and procedural data were analyzed in both groups according to the ablation strategy applied (PVI vs. PVI + substrate modification).

**Results:**

In total, 58 patients (39.4%) had successful ECVs and 89 (60.6%) had failed ECV. Preprocedural characteristics were similar in both groups. Compared to the ECV failure group, patients with successful ECV presented less frequently (34% vs. 60%; *P* = 0.004) and less extended (21.3 ± 22.2% vs. 38.9 ± 27.4% of LA surface, *P* = 0.008) low-voltage areas. Over 55 ± 19 weeks of follow-up, AF-free survival was similar in both groups (72.7% vs. 67.8%, *p* = 0.39). PVI alone resulted in 83% AF-free survival among patients in the ECV successful group at 13 months.

**Conclusion:**

In approximately 40% of patients with PsAF, sinus rhythm can be restored by ECV and maintained for at least 1 month prior to catheter ablation. This clinical response is associated with less abnormal substrate as identified by left atrial voltage mapping and a procedural success rate of >80% with PVI alone.

## Introduction

In unselected patients with persistent atrial fibrillation (PsAF) pulmonary vein isolation (PVI) results in maintenance of sinus rhythm (SR) at one year in approximately 50% of cases (1–3). Currently, there is no established means of predicting in whom PVI alone will be successful and in whom additional ablation strategies may be required. Accurately discriminating between these two populations pre-procedurally would be of considerable value given that PVI is a relatively straightforward and safe procedure whereas ablation strategies beyond PVI can entail more complex approaches and increased risks.

Restoring and maintaining SR with electrical cardioversion (ECV) (4) or antiarrhythmic drugs (AADs) (5–8) prior to catheter ablation has been linked to a favorable response to PVI in patients with PsAF. If true, whether sustained SR prior to catheter ablation mediates or simply acts as a marker for PVI success is unknown, but this association may identify patients in whom PVI alone is effective. We sought to test this hypothesis by retrospectively examining whether restoring SR for at least four weeks prior to catheter ablation predicted ablation procedural success with PVI alone among patients with PsAF.

## Matherials and methods

### Study population

Consecutive patients with PsAF admitted to the Service de Cardiologie, Hôpital Privé Les Franciscaines, Nîmes, France for catheter ablation between January 2021 and April 2022 were retrospectively analyzed.

Exclusion criteria included an age <18 or >85 years, active malignancy, active hyperthyroidism, hypertrophic cardiomyopathy, left ventricular ejection fraction <40%, the presence of a mechanical or bioprosthetic valve, presence of a left atrium (LA) thrombus, a contraindication to anticoagulation, transient ischemic attack or stroke within the preceding 6 months, active cardiac ischemia, ECV performed <4 weeks before catheter ablation, and an inability to restore SR after 3 ECV attempts. The use of AADs was at the discretion of treating physicians. AADs were not discontinued before catheter ablation.

The study was approved by the local ethics committee and all patients provided informed and signed consent.

### Design of the study

**Figure 1:**
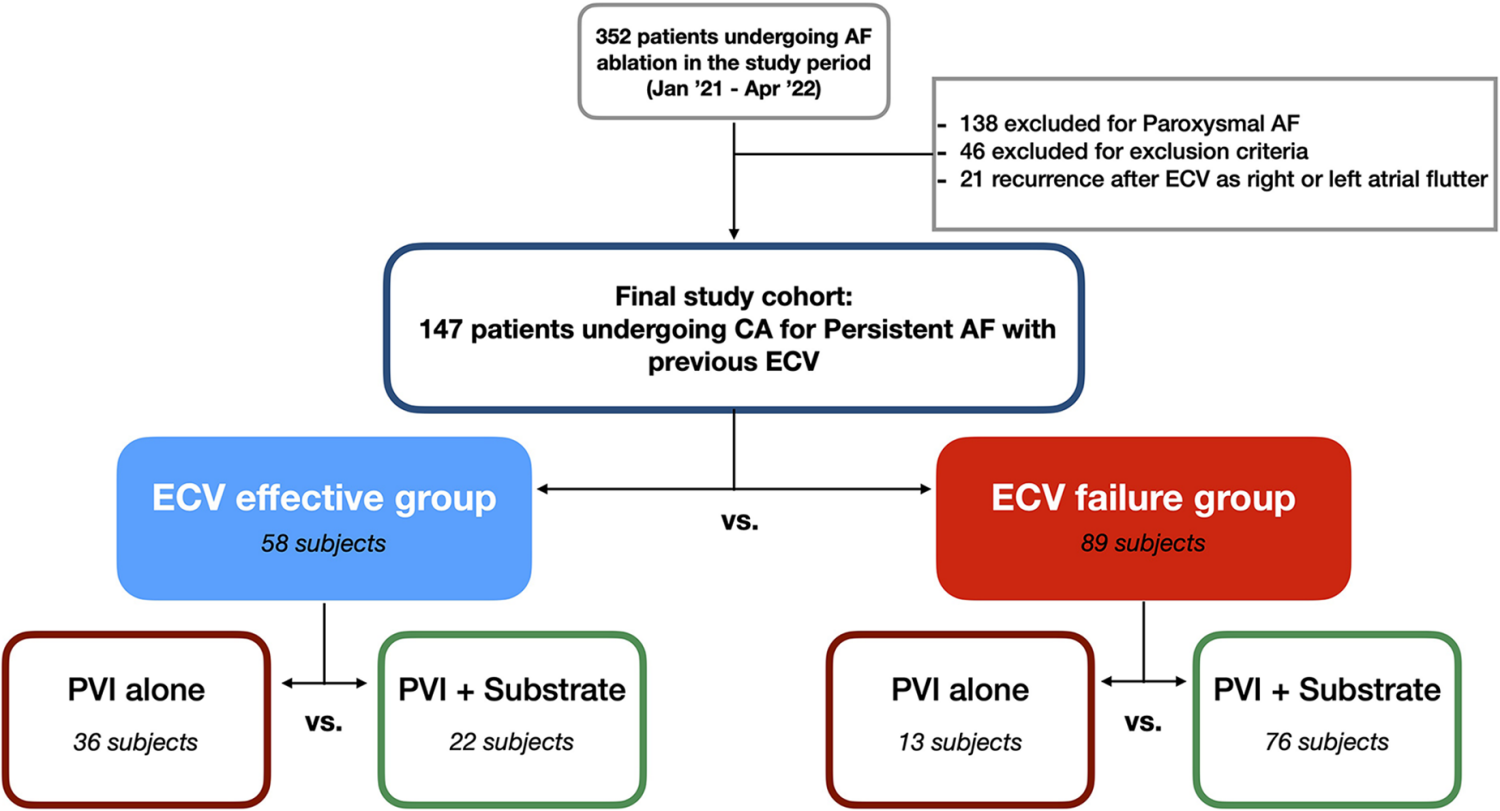
Consort flow diagram showing the enrollment of subjects in the present analysis.

All patients had symptomatic PsAF and underwent pre-ablation elective ECV for symptom management as per protocol at our center. All patients who arrived at the EP laboratory for catheter ablation in SR were included in the group “ECV effective” whereas those in AF formed the group “ECV failure” (Figure 1).

### Procedural aspects

Ablation procedures were carried out under general anesthesia and on uninterrupted direct oral anticoagulant or vitamin K antagonist (international normalized ratio 2.0–3.0). Transesophageal echocardiography was systematically performed to exclude the presence of intracardiac thrombi and to guide transseptal puncture. Before LA access was obtained, intravenous heparin was administered to achieve an activated clotting time >350 s throughout the entire procedure.

Ablation procedures were conducted under electronatomical navigation system guidance (CARTO 3, Biosense Webster, Diamond Bar, CA) using a multipolar diagnostic catheter (Pentaray, Biosense Webster) and an open irrigated contact force-sensing ablation catheter (SmartTouch SF, Biosense Webster). Before reconstruction of LA anatomy, ECV was performed to obtain analysis of endocardial voltage during SR in all patients. A second ECV was performed after PVI in the case SR was not restored or AF was triggered again during mapping.

Low voltage areas (LVAs) were defined as regions of the LA with endocardial bipolar voltage <0.5 mV. In patients with previous AF ablation, PVs antra were not included in the amount of LVA.

**Figure 2:**
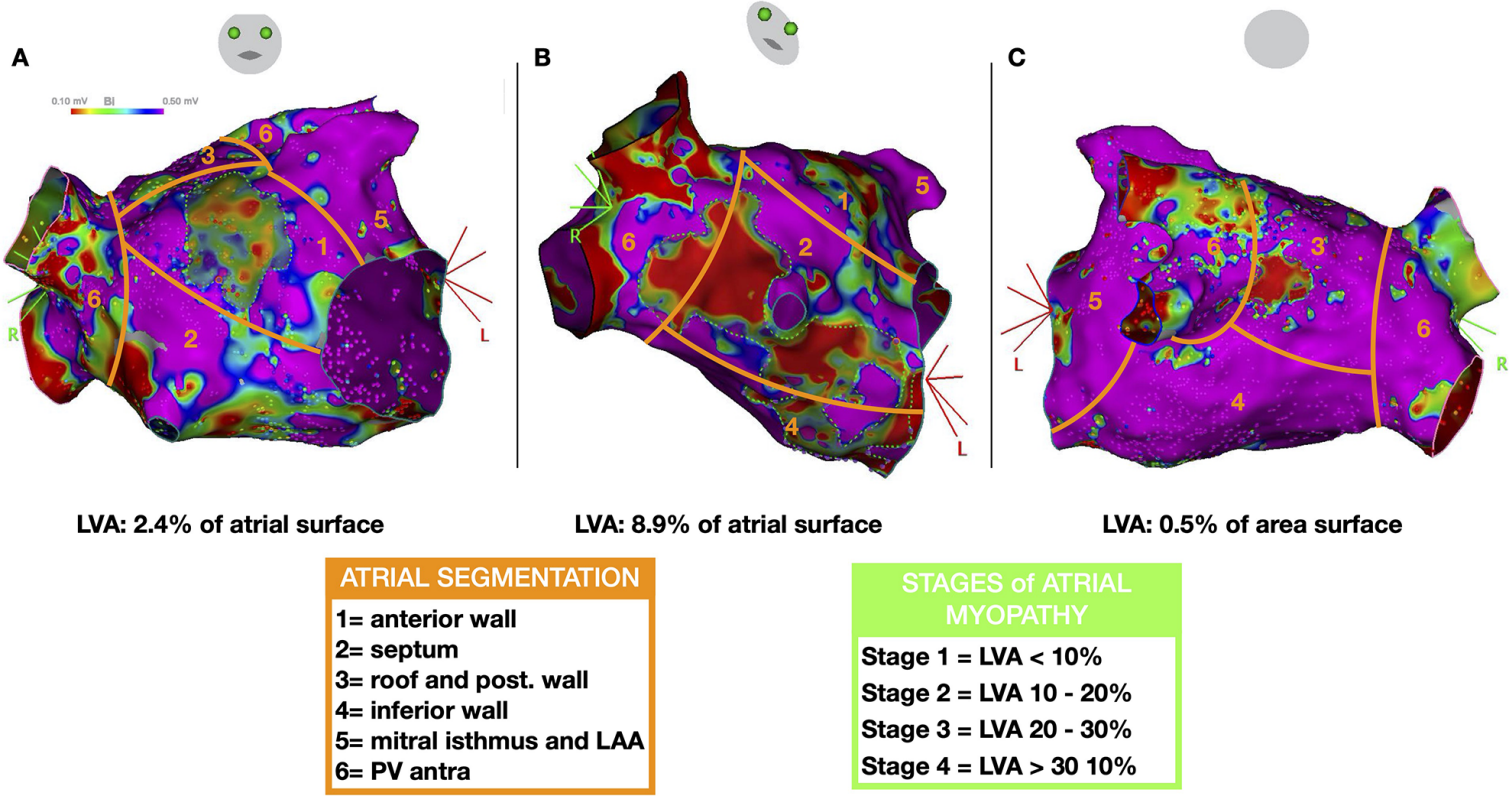
Left atrial segmentation to characterize the distribution of low voltage areas (LVA) and stages of atrial myopathy. Representative case of a patient with LVAs: cranial right anterior oblique [RAO, **(A)**], RAO **(B)** and caudal postero-anterior views **(C)** demonstrating three LVAs on the anterior, septal, and posterior walls (delineated by green dotted lines) encompassing 11.5% of the left atrial surface area (classified as stage 2 atrial myopathy).

The total surface of the LVA was indexed to the total surface of the LA and classified into 4 stages of atrial myopathy as reported in the DECAAF study (Figure 2) (9, 10).

Radiofrequency lesions (RF) were delivered as previously reported (11–13). The energy power used was 50W except in the great cardiac vein (GCV) where it was 25W. The ablation indices targeted were 250 inside the GCV, 350 on the posterior aspects of the LA, and ≥450 elsewhere. All procedures were performed by two experienced operators (GL, AB) and the ablation strategies used (PVI alone, Marshal-PLAN, or PVI + LVA isolation) was determined by operator preference (14, 15).

Mitral line block was obtained using the following steps in all the patients, as previously described (14). After CS cannulation using a steerable sheath, iodine contrast was injected in the proximity of the Vieussens valve through a left internal mammary artery catheter to localize the VOM. An angioplasty guidewire and subsequently a preloaded angioplasty balloon (length 6–8 mm, nominal diameter 2–3.5 mm) was dilatated at 3–5 atm in the proximal VOM to occlude its lumen. A contrast injection in the balloon lumen was performed to visualize VOM tributary branches before the injection of 8–10 ml of 96% ethanol (3 separate 1 min injections 3 ml). Ethanol infusion was considered successful if repeat angiograms between the 3 injections demonstrated (1) stability of the angioplasty balloon; (2) visualization of the distal VOM arborization; (3) absence of contrast leakage back in the CS; (4) absence or limited dissection of the VOM; and (5) progressive appearance of tissue contrast staining. In all the patients after VOM alcoholization, a new endocardial electroanatomic map of the mitral isthmus region was acquired to visualize the effect of VOM alcoholization and RF ablation was performed (of the “saddle” area between the high ridge, left atrial appendage, left superior pulmonary vein) targeting endocardial insertion site of Marshall bundles and completing an endocardial mitral line from left inferior PV to mitral annulus. RF ablation was also performed in the CS targeting epicardial muscular bundles identified by sharp near-field signals, in correspondence with endocardial scar resulting from VOM alcoholization and avoiding RF delivery in the proximal CS. Mitral isthmus block will be validated in SR by pacing maneuvers.

### Follow-up

Follow-up visits were performed in an outpatient clinic at 3, 6, and 12 months after the index ablation procedure. When treating physicians prescribed AADs at hospital discharge, they were maintained for 4 weeks after the procedure.

Subsequently, follow-up visits occurred once yearly. Clinical examination, an ECG, and a 48 h Holter were performed at each visit in addition to any investigations initiated in response to patient symptoms suggestive of AF recurrence. AF recurrence was defined as any documented episode of AF lasting longer than 30 s after a 90-day blanking period post-procedure.

### Statistical analysis

Continuous variables are expressed as mean value ± standard deviation or median value with interquartile range, as appropriate. Categorical variables are presented as number and percentages, and their proportions were compared using a Chi-square analysis or Fisher's exact tests, as appropriate. Continuous variables were compared using Student *t*-tests for independent samples or Mann–Whitney *U* tests, depending on data normality.

Clinical variables associated with ECV group as well as LVAs were identified using logistic regression and are presented as odds ratio point estimates with 95% confidence intervals. Univariate and multivariate analyses were performed with the Cox proportional hazard regression model to identify variables associated to AF recurrences. Variables identified as significant based on *p* < 0.1 in univariable analyses were included in a multivariable model. Kaplan–Meier curve and log-rank test were used to assess AF-free survival during the whole follow-up. Data were analyzed with SPSS software version 26.0 (SPSS Inc., Chicago, Illinois).

## Results

### Patient characteristics

In total, 147 consecutive patients were included (Table 1). Their mean age was 70.1 ± 9.4 years and 98 (66.7%) were male. PVI alone was performed in 49 patients (33.3%), 8 of whom had LVAs identified on the day of the procedure (16.3%). Additional substrate ablation was performed in the remaining 98 patients (66.7%), 63 of whom had LVAs (64.3%).

**Table 1:** Baseline patient characteristics.

	Overall *N =* 147	ECV effective *N =* 58 (39.5%)	ECV failure *N* = 89 (60.5%)	*P*
Male sex	98 (66.7)	39 (67.2)	59 (66.3)	0.1
Age (years)	70.1 ± 9.4	71 ± 9.1	69 ± 9.7	0.08
LVEF (%)	52.3 ± 9.9	50.7 ± 9.5	53.3 ± 10.5	0.13
LA volume (ml)	143.2 ± 34.6	136 ± 37.5	147 ± 32.2	0.14
LAVI (ml/m^2^)	71.1 ± 23.3	65.5 ± 25.9	74.7 ± 20.8	0.02
Duration of AF history (months)	49.7 ± 54.4	49.2 ± 51.3	49.9 ± 56.5	0.9
Time from ECV to catheter ablation (weeks)	10.4 ± 7.5	9.2 ± 6.1	11.2 ± 8.3	0.11
Comorbidities				
Hypertension	111 (75.5)	45 (77.6)	66 (74.2)	0.69
Dyslipidemia	42 (28.6)	19 (32.8)	23 (25.8)	0.45
Diabetes mellitus	32 (21.8)	15 (5.9)	17 (19.1)	0.41
Tobacco use	30 (20.4)	12 (20.7)	18 (20.2)	1.0
History of stroke	14 (9.5)	7 (12.1)	7 (7.9)	0.41
History of coronary artery disease	41 (27.9)	18 (31.0)	23 (25.8)	0.57
Obstructive sleep apnea	30 (20.4)	15 (25.9)	15 (16.9)	0.21
Peripheral vascular disease	14 (9.5)	3 (5.6)	11 (12.4)	0.24
History of Tachycardia-induced cardiomyopathy	44 (29.9)	22 (37.9)	22 (24.7)	0.1
Overweight	34 (23.1)	13 (22.4)	21 (23.6)	1.0
Body mass index (kg/m^2^)	29.2 ± 4.9	29.5 ± 4.7	28.9 ± 5.1	0.41
CHA_2_DS_2_-VASc score	3.0 ± 1.5	3.1 ± 1.5	2.8 ± 1.4	0.02
Moderate valvular disease	22 (15.0)	9 (15.5)	13 (14)	1.0
Previous AF ablation	42 (28.6)	17 (29.3)	25 (28.1)	1.0
Reconnected PV^a^	15 (35.7)	7 (41.2)	6 (24.0)	0.1
Previous CTI ablation	8 (5.4)	3 (5.2)	5 (5.6)	1.0
Procedural characteristics				
Presence of LVA	72 (49.7)	18 (31.0)	54 (60.7)	0.0001
LVA area (%)	34.7 ± 26.9	21.3 ± 22.1	38.9 ± 27.4	0.008
Atrial myopathy stage				
1	84 (57.1)	43 (74.1)	41 (46.1)	0.001
2	18 (12.2)	7 (12.1)	11 (12.4)	
3	11 (7.5)	4 (6.9)	7 (7.9)	
4	34 (23.1)	4 (6.9)	30 (33.7)	
Localization of LVA				
Roof-posterior	46 (63.8)	8 (44.4)	38 (70.4)	0.08
Anterior	48 (66.6)	12 (66.7)	26 (48.1)	0.28
Inferior	18 (25.0)	1 (5.5)	17 (31.4)	0.03
Lateral	7 (16.7)	3 (16.7)	4 (7.4)	0.35
Septum	17 (23.1)	1 (5.5)	16 (29.6)	0.05
Atrial tachycardia during the procedure	8 (5.4)	3 (5.2)	5 (5.6)	0.61
**PVI alone**	49 (33.3)	36 (62.1)	13 (14.6)	0.0001
Anti-arrhythmic medications before the procedure				
Beta-blockers	40 (27.2)	18 (31.0)	22 (24.7)	0.45
Class 1C-3	48 (32.6)	20 (34.5)	28 (31.5)	0.72
Anti-arrhythmic medications during follow-up				
Class 1C-3	35 (30.6)	13 (22.4)	32 (36.0)	0.1

Data are presented as *N*.(%) or mean ± SD.

AF, atrial fibrillation; ECV, electrical cardioversion; CTI, cavo-tricuspid isthmus; LA, Left atrium; LAVI, left atrium volum indexed for body surface area; LVA, low-voltage areas LVEF, left ventricular ejection fraction; PVI, pulmonary vein isolation.

^a^ Percentage calculated on the number of REDO patients.

Nine minor complications (6.1%) occurred (4 groin hematomas, 1 arteriovenous fistula and 4 pericarditis). There were no cases of cardiac tamponade, stroke-transient ischemic attack, atrioesophageal fistula, or death.

ECV was performed a mean of 10.4 ± 7.4 weeks before ablation procedures. On the day of the ablation procedure, 58 patients (39.4%) were in SR (*ECV effective*) whereas 89 patients (60.6%) were in AF (*ECV failure*). CHA_2_DS_2_-VASc scores were higher but LAVI values were lower in the ECV effective than in the ECV failure group (3.1 ± 1.5 vs. 2.8 ± 1.4, *P* = 0.02; and 65.5 ± 25.9 vs. 74.7 ± 20.8 ml/m^2^, *P* = 0.02; respectively). In the multivariable regression model, CHA_2_DS_2_-VASc scores (OR 1.39, 1.08–1.79) and presence of low-voltage areas (0.29, 0.14-0.62) predicted the ECV Efficacy group (Supplementary Table S1).

A previous AF ablation was reported in 42 subjects (28.6%) with a rate of reconnected pulmonary veins of 41.2% in the ECV effective and 24.0% in the ECV failure groups (*p* = 0.1).

LVAs were more frequently found in the ECV failure group (34% vs. 60% of patients; *P* = 0.004) and, when present, they were more extensive when indexed to the total LA surface (21.3 ± 22.2% vs. 38.9 ± 27.4%, *P* = 0.008). Overall, fewer patients in the ECV effective group were classified as having advanced (stage 3 or 4) atrial myopathy (14% vs. 42%, *P* < 0.001). In the multivariable regression model, ECV failure was the only independent predictor of LVA at electroanatomic mapping (OR 3.2, 1.5–6.9; *P* = 0.002) (Supplementary Table S2).

### Ablation strategies

Among the 58 patients in the ECV effective group, 36 underwent PVI alone (62%) and 22 underwent PVI + substrate ablation (38%). Among the remaining 89 patients, 13 (15%) underwent PVI alone and 76 (85%) underwent PVI + substrate ablation (Table 2). Marshall-PLAN approach was more frequently executed in patients in the ECV failure group. As reported in Table 3, substrate ablation strategies included Marshall-PLAN approach (which included a posterior box in all the patients) in 4 (18.2%) and 45 (59.2%), respectively. A posterior box and an anterior line targeting LVAs were respectively executed in 10 and 9 patients in the ECV effective group and in 19 and 10 patients in the ECV failure groups.

**Table 2:** Set of procedural lesions performed in the substrate groups among ECV effective and ECV failure groups.

	ECV effective (*N*. 22)	ECV failure (*N*. 76)	*P*-value
Posterior box	14 (63.6)	64 (84.2)	0.06
Mitral isthmus block	4 (18.2)	45 (59.2)	0.001
*Success rate of VOM* ^a^	3 (75.0)	36 (80.0)	1.000
Anterior line	9 (40.9)	10 (13.1)	0.01
CTI	6 (27.7)	6 (7.8)	0.02

VOM, vein of marshal ethanol infusion; CTI, cavo-tricuspid isthmus ablation.

^a^ Calculated on the total of attempted VOM alcoholizations.

**Table 3:** Characteristics of the population according to group and procedures.

	ECV effective			ECV failure		
	PVI alone	Substrate	*P*	PVI alone	Substrate	*P*
No (%)	36 (62.1)	22 (37.1)		13 (14.6)	76 (85.4)	
Male sex	24 (66.7)	15 (68.1)	1.0	7 (53.8)	52 (68.4)	0.35
Age	71.3 ± 10.5	72.4 ± 8.5	0.68	69.4 ± 9.0	68.9 ± 9.3	0.89
LVEF	50.4 ± 11.5	51.1 ± 9.1	0.83	54.8 ± 6.5	53.1 ± 9.9	0.58
LA volume (ml)	141.6 ± 40.4	128.8 ± 31.4	0.22	126.5 ± 31.0	150.8 ± 31.2	0.1
LAVI (ml/m^2^)	69.0 ± 26.6	59.9 ± 24.3	0.19	61.6 ± 24.0	77.0 ± 19.5	0.04
LAVI > 70 ml/m^2^	13 (36.1)	9 (40.9)	0.78	3 (23.1)	46 (60.5)	0.01
BMI (kg/m^2^)	28.6 ± 4.3	31.2 ± 5.1	0.06	30.6 ± 5.7	28.6 ± 5.0	0.25
Time between ECV and ablation	67.5 ± 14.9	84.1 ± 35.4	0.52	84.7 ± 80.0	77.5 ± 53.8	0.67
AF duration (months)	29.7 ± 29.6	80.8 ± 63.1	0.0001	35.4 ± 38.9	52.5 ± 58.9	0.31
Prior AF ablation (%)	5 (13.9)	12 (54.5)	0.002	0 (0)	25 (32.9)	0.02
History of CAD	23 (25.8)	18 (31.0)	1.0	3 (23.1)	20 (26.3)	1.0
LVA	6 (17.1)	12 (57.1)	0.003	2 (15.4)	51 (67.1)	0.001
– *LVA not targeted* – *LVA completely targeted* – *LVA partially targeted*	5 (83.3)	1 (8.3)		2 (100)	10 (19.6)	
	0	6 (50.0)	0.0001	0	12 (23.5)	0.002
	1 (16.7)	5 (41.7)		0	29 (56.9)	
LA-indexed LVA area (%)	20.0 ± 18.9	21.9 ± 24.2	0.86	40.0 ± 42.4	38.8 ± 27.3	0.95
Atrial myopathy (stage)						
1	32 (88.9)	11 (50.0)	0.007	11 (84.6)	30 (39.5)	0.004
2	1 (2.8)	6 (27.3)		1 (7.7)	10 (13.2)	
3	2 (5.6)	2 (9.1)		0	7 (9.2)	
4	1 (2.8)	3 (13.6)		1 (7.7)	29 (38.2)	
Atrial tachycardia	0	3 (13.6)	0.05	0	5 (6.6)	0.44
Procedural duration	71.3 ± 13.0	100.6 ± 28.5	0.02	71.3 ± 13.0	100.6 ± 28.5	0.0001
RF ablation duration	899.3 ± 298.8	713.7 ± 434.8	0.07	862.3 ± 205.0	1,052.1 ± 424.4	0.02
Complications	1 (2.8)	4 (18.2)	0.06	1 (7.7)	3 (3.9)	0.47

Data are presented as *N*. (%) or mean ± SD.

AF, atrial fibrillation; ECV, electrical cardioversion; CTI, cavo-tricuspid isthmus; LA, Left atrium; LAVI, left atrium volum indexed for body surface area; LVA, low-voltage areas; LVEF, left ventricular ejection fraction; PVI, pulmonary vein isolation.

Among patients in the ECV effective group, substrate modification beyond PVI was more often performed in patients with longer AF durations (80.8 ± 63.1 vs. 29.7 ± 29.7 months, *P* = 0.002), with LVAs (57% vs. 17%, *P* = 0.003), and previous AF ablation (55% vs. 14%, *P* = 0.002). Among patients in the ECV failure group, substrate modification was more often performed in patients with larger LAVIs (77.1 vs. 61.6 ml/m^2^, *P* = 0.04), with LVAs (67% vs. 15%, *P* = 0.001), and previous AF ablation (33% vs. 0%, *P* = 0.02).

### Clinical outcomes

**Figure 3:**
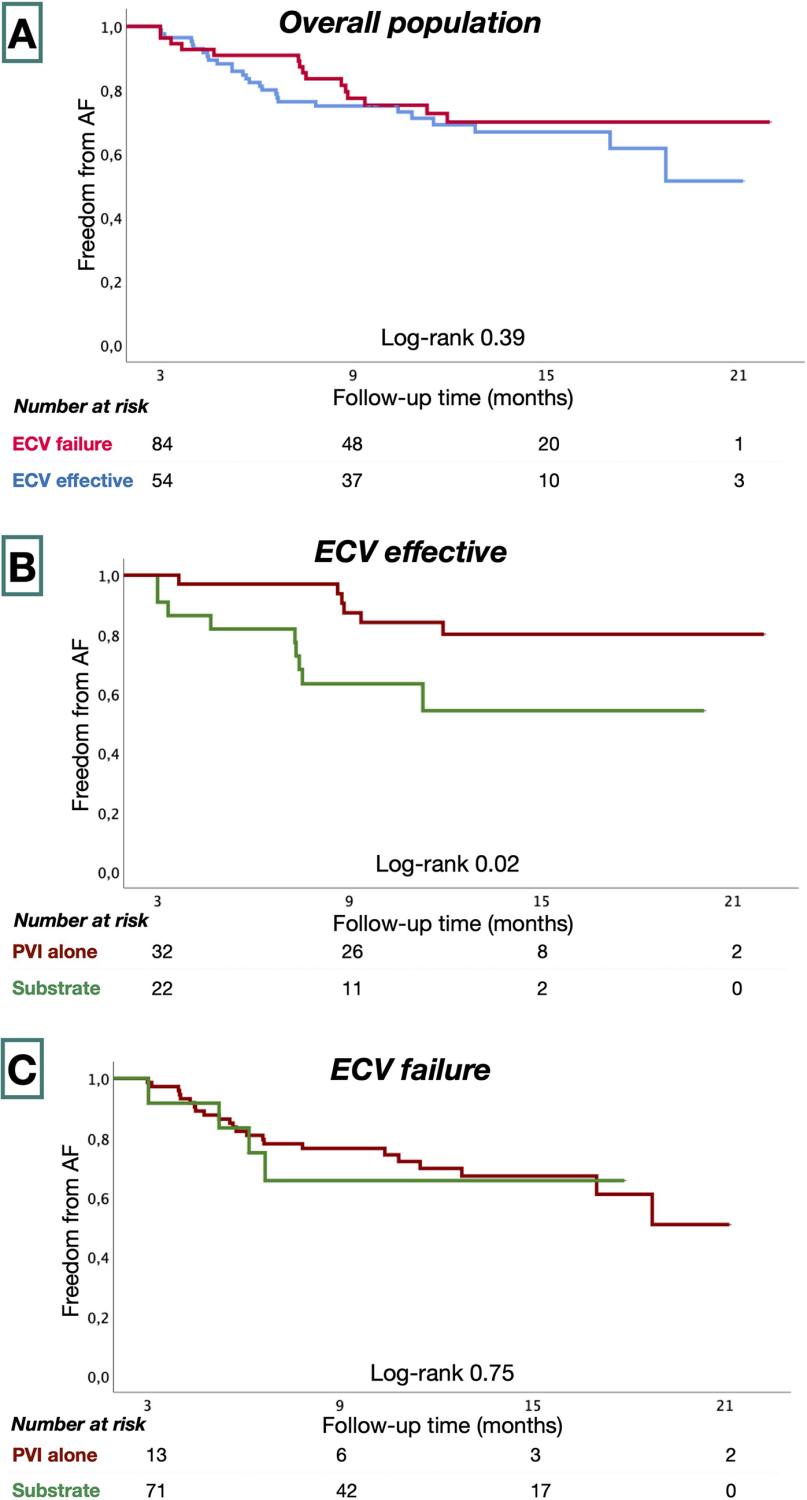
Kaplan–Meier survival estimates from AF recurrence in the overall study population **(A)**, ECV effective **(B)**, and ECV failure groups **(C)**.

Patients were followed for 55 ± 19 weeks following ablation procedures. Eight patients (5.4%) had incomplete follow-up. Forty-two patients (30%) had recurrent AF. AF-free survival was similar in ECV effective and in the ECV failure groups (73% vs. 68%, *P* = 0.39) at 58.8 ± 18.3 and 52.4 ± 18.6 weeks, respectively (Figure 3). In the ECV effective group, patients treated with PVI alone had greater AF-free survival than those who underwent additional substrate ablation (83% vs. 59%; *P* = 0.02). In the ECV failure group, AF-free survival was similar among patients treated with PVI and with additional substrate ablation (64% vs. 70%, *P* = 0.75).

Patients undergoing their first procedure presented better AF-free survival compared to subjects undergoing a REDO (Supplementary Figure S1). At multivariate analysis (Table 4), LVA not targeted by ablation was the strongest predictor of AF recurrences (HR 1.39, 95CI: 0.97–1.98; *p* = 0.07).

**Table 4:** Univariate and multivariate predictors of AF recurrences.

	Univariate				Multivariate			
			95 CI				95 CI	
	*P* value	HR	lower	upper	*P* value	HR	lower	upper
ECV working	0.39	1.31	0.70	2.47				
LAVI	0.73	1.00	0,99	1,02				
LA dimensions <70 ml/m^2^	0.12	1.64	0.87	3.08				
LVEF	0.60	1.01	0.98	1.04				
Age	0.36	1.01	0.98	1.05				
Female sex	0.16	156	0.83	2.93				
DM	0.21	1.56	0.78	3.13				
LVA	0.09	1.73	0.91	3.29	0.57	1.15	0.69	1.93
LVA not targeted	0.02	2.04	1.11	3.76	0.07	1.39	0.97	1.98
Atrial myopathy stage	0.11	1.21	0.96	1.52				
CAD	0.02	2.77	1.18	6.58	0.09	1.39	0.94	2.04
CHADS-VASc score	0.79	1.02	0.83	1,26				
OSAS	0.18	0.53	0.21	1.35				
HF	0. 78	0.89	0.39	2.01				
AF duration <24 months	0.13	0.60	0.31	1.17				
Previous AF ablation	0.003	2.55	1.37	4.74	0.50	1.15	0.78	1.68

ECV, electrical cardioversion; AF, atrial fibrillation; LAVI, left atrium volum indexed for body surface area; LVEF, left ventricular ejection fraction; LVA, low-voltage areas; CAD, coronary artery disease; OSAS, obstructive sleep apnea syndrome; HF, heart failure.

## Discussion

### Main findings

The present study sought to assess the response to ECV as a potential tool to identify patients with PsAF who might respond favorably to PVI alone. The principal findings of this study are as follows: (1) In an unselected cohort of patients with PsAF, SR could be restored and maintained in 40% for ≥4 weeks prior to catheter ablation. (2) The predictive ability of the clinical variables tested in this study for ECV success was poor. (3) Patients in whom ECV resulted in sustained SR were less likely to have LVA detected on endocardial mapping and, when LVAs were present, were less extensive than in patients in whom AF recurred. (4) PVI alone resulted in SR maintenance of 83% at 1 year in the ECV effective group and 64% in the ECV failure group.

### Response to ECV to stratify patients with psAF

Patients with PsAF are characterized by considerable variability in their degree of atrial remodeling (10, 16). The presence of LVAs has been proposed as a marker of more advanced atrial remodeling and has been linked to poor results from PVI alone (15, 17–19).

In our study, the presence and extent of LVAs were almost twice as great in patients in whom SR could not be maintained prior to catheter ablation than in those in whom SR was maintained and ECV failure was the only independent predictor of LVAs in our analysis.

Of importance, in the cohort here analyzed patients belonging to the ECV effective group could not be identified based on pre-procedural available parameters.

If maintenance of sustained SR prior to catheter ablation is associated with less extensive substrate abnormalities in patients with PsAF, it may serve pre-procedurally to identify individuals in whom PVI alone is sufficient.

This is supported by previously published data. In a large retrospective analysis, Eberly et al. found that PVI led to excellent long-term outcomes in patients with PsAF in whom SR was stably restored prior to catheter ablation (8). In a study of catheter ablation for PsAF targeting LVAs, PVI alone led to AF-free survival of 84% at 13 months in the subgroup of patients without LVAs and in whom SR was stably restored after ECV (4). Similarly, in two prospective single-center studies, the clinical response to AADs identified patients with PsAF who were more likely to respond to PVI (5, 7). Notably, however, not all studies examining the clinical response to ECV or AAD have found it to be predictive of PVI success (20).

Whether maintenance of SR mediates or is simply a marker of PVI effectiveness in patients with PsAF is unknown. However, there are data to suggest that it may not simply reflect less extensive extrapulmonary vein abnormalities. Benák et al. observed that patients experiencing a “step-back” to a paroxysmal AF phenotype with amiodarone had satisfactory long-term success from PVI alone (6). Rivard et al. reported that SR restoration by ECV before PsAF catheter ablation was associated with less extensive ablation required to restore SR when ablating during spontaneous or induced AF with similar long-term clinical success rates. On these bases the authors postulated that SR maintenance prior to catheter ablation may reverse adverse atrial remodeling (21).

For instance, the adoption in our study of a minimum of time of 4 weeks between ECV and catheter ablation is explained by the fact that reverse electrical remodeling in clinical and experimental models of chronic AF requires at least 4 weeks to take place (21–24).

### PVI alone for patients with persistent AF

The 1-year success rate of PVI alone in unselected patients with PsAF is approximately 50%–60% (16). Accordingly, ablation strategies beyond PVI have been developed, many of which incorporate targeting presumably abnormal substrate and have been reported to yield favorable results (14, 15, 25). These approaches included the Marshall-PLAN and PVI + LVAs ablations which aims at making fibrillatory conduction less probable through transecting conduction through atrial *isthmi* or eliminating conduction throughout diseased areas (14, 15, 25). These approaches have been adopted in the present series according to operator choice. However, whether all patients with PsAF benefit from these more extensive ablation approaches is less clear, but it is conceivable that PVI alone may be sufficient for a sizeable proportion of patients with “earlier” stages of atrial remodeling. Our data suggest this to be the case, as patients with a favorable response to pre-ablation ECV exhibited AF-free survival of 83% with PVI alone at 13 months. These patients, although meeting criteria for PsAF, had shorter AF durations and were less likely to have LVAs (and when LVAs were present they were less extensive) when compared to their counterparts in whom AF recurred following ECV.

Since this is true also when comparing patients in the ECV effective group undergoing PVI alone or substrate modulations procedures. It is thus presumable that other parameters too, like substrate evaluation which can be in turn related to history of AF duration or previous catheter ablations, could play a role in selecting ablation strategy. However, taken altogether, our results suggest that assessing the results of ECV after an adequate period (i.e., 4–6 weeks) (22, 23) in a population with PsAF may represent a valuable pre-procedure tool to identify the healthiest patients of persistent AF spectrum with less diseased atria who may benefit from a PVI only ablation strategy. This information could be of pivotal importance for procedural planning considering the recent emerging of new technologies and energies for PVI among patients with PsAF (26, 27).

### Limitations

The retrospective, single-center, and non-randomized nature of the study is an important limitation of this analysis. The risk of selection bias exists whereby “healthier” patients were treated with PVI alone, which is supported by the difference in the proportion of patients who underwent PVI in the ECV success and ECV failure groups (62% vs. 15%). However, this does not negate the conclusion that a subset of patients with PsAF can be effectively treated with PVI alone nor that the clinical response to ECV prior to ablation can help identify such “healthier” patients. The limited dimensions of the present study do not permit to make firm conclusions on populations subgroups, like patients undergoing a REDO procedure.

This study is not aimed at evaluating specific substrate ablation strategies beyond PVI and therefore this objective is beyond the aims of the present study. LVA-based ablation strategies have been more robustly evaluated by others still yielding conflicting results to date (3, 15, 18, 28–32). Since data of PsAF recurrence after ECV was collected as a dichotomic variable at admission, we can not provide information regarding timing of recurrence.

Finally, our results may not be generalizable to the full spectrum of patients with PsAF given that our exclusion criteria included potentially important variables such as a low left ventricular ejection fraction and the presence of a prosthetic valve. Given these limitations, our study should be considered hypothesis-generating. Our hypothesis is currently being evaluated in the *Pulmonary vein isolation Alone or in Combination wIth substrate modulation aFter electrIcal Cardioversion failure in patients with persistent atrial fibrillation* (PACIFIC) *study.* This multicenter, prospective, randomized study will use patients’ clinical response to ECV to guide adjunctive ablation strategies beyond PVI (24).

## Conclusions

In our study, in approximately 40% of patients with PsAF SR was stably restored by ECV for at least 1 month prior to catheter ablation. This subgroup of patients tended to have evidence of less extensive atrial remodeling as defined by voltage mapping and exhibited AF-free survival exceeding 80% at 13 months with PVI alone. We could not identify strong clinical predictors of ECV success. Among patients with PsAF planned to undergo catheter ablation, the response to ECV may be a simple method to identify those in whom PVI alone is sufficient.

## Data Availability

The raw data supporting the conclusions of this article will be made available by the authors, without undue reservation.

## References

[1] Boveda S Metzner A Nguyen DQ Chun KRJ Goehl K Noelker G Single-procedure outcomes and quality-of-life improvement 12 months post-cryoballoon ablation in persistent atrial fibrillation: results from the multicenter CRYO4PERSISTENT AF trial. JACC Clin Electrophysiol. (2018) 4:1440–7. 10.1016/j.jacep.2018.07.007 30466850

[2] Tondo C Iacopino S Pieragnoli P Molon G Verlato R Curnis A Pulmonary vein isolation cryoablation for patients with persistent and long-standing persistent atrial fibrillation: clinical outcomes from the real-world multicenter observational project. Heart Rhythm. (2018) 15:363–8. 10.1016/j.hrthm.2017.10.038 29107190

[3] Verma A Jiang C-y Betts TR Chen J Deisenhofer I Mantovan R Approaches to catheter ablation for persistent atrial fibrillation. N Engl J Med. (2015) 372:1812–22. 10.1056/NEJMoa1408288 25946280

[4] Jadidi AS Lehrmann H Keyl C Sorrel J Markstein V Minners J Ablation of persistent atrial fibrillation targeting low-voltage areas with selective activation characteristics. Circ Arrhythm Electrophysiol. (2016) 9(3):e002962. 10.1161/CIRCEP.115.002962 26966286

[5] Okawa K Hara S Morimoto T Tsushima R Sudo Y Sogo M Effect of preprocedural pharmacologic cardioversion on pulmonary vein isolation in patients with persistent atrial fibrillation. Heart Rhythm. (2021) 18:1473–9. 10.1016/j.hrthm.2021.04.027 33932587

[6] Benák A Kohári M Herczeg A Makai A Bencsik G Sághy L Selecting persistent atrial fibrillation patients for pulmonary vein isolation based on the response to amiodarone: efficacy of the «one step back» strategy. J Interv Card Electrophysiol. (2019) 56:291–7. 10.1007/s10840-019-00524-z 30820779

[7] He SN Tian Y Shi L Wang YJ Xie BQ Li XX Identification of circumferential pulmonary vein isolation responders among patients with persistent atrial fibrillation: clinical value of the sequential low-dose ibutilide test. Europace. (2020) 22:1197–205. 10.1093/europace/euaa095 32514560

[8] Eberly LA Lin A Park J Khoshnab M Garg L Chee J Presence of sinus rhythm at time of ablation in patients with persistent atrial fibrillation undergoing pulmonary vein isolation is associated with improved long-term arrhythmia outcomes. J Interv Card Electrophysiol. (2023) 66(6):1455–64. 10.1007/s10840-022-01441-4 36525168

[9] Huo Y Gaspar T Pohl M Sitzy J Richter U Neudeck S Prevalence and predictors of low voltage zones in the left atrium in patients with atrial fibrillation. Europace. (2018) 20:956–62. 10.1093/europace/eux082 28605524

[10] Marrouche NF Wilber D Hindricks G Jais P Akoum N Marchlinski F Association of atrial tissue fibrosis identified by delayed enhancement MRI and atrial fibrillation catheter ablation: the DECAAF study. JAMA. (2014) 311:498–506. 10.1001/jama.2014.3 24496537

[11] Bortone A Albenque JP Ramirez FD Haïssaguerre M Combes S Constantin M 90 vs 50-watt radiofrequency applications for pulmonary vein isolation: experimental and clinical findings. Circ Arrhythm Electrophysiol. (2022) 15(4):e010663. 10.1161/CIRCEP.121.010663 35363039

[12] Bortone AA Ramirez FD Constantin M Bortone C Hébert C Constantin J Optimal interlesion distance for 90 and 50 watt radiofrequency applications with low ablation index values: experimental findings in a chronic ovine model. Europace. (2023) 25(11):euad310. 10.1093/europace/euad310 37851513 PMC10629717

[13] Bortone AA Ramirez FD Combes S Laborie G Albenque JP Sebag FA Optimized workflow for pulmonary vein isolation using 90-w radiofrequency applications: a comparative study. J Interv Card Electrophysiol. (2024) 67(2):353–61. 10.1007/s10840-023-01630-9 37639157

[14] Derval N Duchateau J Denis A Ramirez FD Mahida S André C Marshall bundle elimination, pulmonary vein isolation, and line completion for ANatomical ablation of persistent atrial fibrillation (Marshall-PLAN): prospective, single-center study. Heart Rhythm. (2021) 18:529–37. 10.1016/j.hrthm.2020.12.023 33383226

[15] Huo Y Gaspar T Schönbauer R Wójcik M Fiedler L Roithinger FX Low-voltage myocardium-guided ablation trial of persistent atrial fibrillation. NEJM Evid. (2022) 1(11):EVIDoa2200141. 10.1056/EVIDoa2200141 38319851

[16] Kirchhof P Calkins H. Catheter ablation in patients with persistent atrial fibrillation. Eur Heart J. (2017) 38:20–6. 10.1093/eurheartj/ehw260 27389907 PMC5353871

[17] Vlachos K Efremidis M Letsas KP Bazoukis G Martin R Kalafateli M Low-voltage areas detected by high-density electroanatomical mapping predict recurrence after ablation for paroxysmal atrial fibrillation. J Cardiovasc Electrophysiol. (2017) 28:1393–402. 10.1111/jce.13321 28884923

[18] Yang G Zheng L Jiang C Fan J Liu X Zhan X Circumferential pulmonary vein isolation plus low-voltage area modification in persistent atrial fibrillation. JACC Clin Electrophysiol. (2022) 8:882–91. 10.1016/j.jacep.2022.03.012 35863814

[19] Bergonti M Spera FR Ferrero TG Nsahlai M Bonomi A Tijskens M Characterization of atrial substrate to predict the success of pulmonary vein isolation: the prospective, multicenter MASH-AF II (multipolar atrial substrate high density mapping in atrial fibrillation) study. J Am Heart Assoc. (2023) 12(1):e027795. 10.1161/JAHA.122.027795 36565183 PMC9973584

[20] Yadav R Brilliant J Akhtar T Milstein J Sampognaro JR Marine J Relationship between amiodarone response prior to ablation and one-year outcomes of catheter ablation for atrial fibrillation. J Cardiovasc Electrophysiol. (2023) 34:860–8. 10.1111/jce.15845 36738148

[21] Rivard L Hocini M Rostock T Cauchemez B Forclaz A Jadidi AS Improved outcome following restoration of sinus rhythm prior to catheter ablation of persistent atrial fibrillation: a comparative multicenter study. Heart Rhythm. (2012) 9:1025–30. 10.1016/j.hrthm.2012.02.016 22342863

[22] Everett TH 4th Li H Mangrum JM McRury ID Mitchell MA Redick JA Electrical, morphological, and ultrastructural remodeling in a canine model of chronic atrial fibrillation. Circulation. (2000) 102:1454–60. 10.1161/01.CIR.102.12.1454 10993867

[23] Chalfoun N Harnick D Pe E Undavia M Mehta D Gomes JA. Reverse electrical remodeling of the atria post cardioversion in patients who remain in sinus rhythm assessed by signal averaging of the P-wave. Pacing Clin Electrophysiol. (2007) 30:502–9. 10.1111/j.1540-8159.2007.00700.x 17437574

[24] Bortone AA Marijon E Limite LR Lagrange P Brigadeau F Martins R Pulmonary vein isolation alone or in combination with substrate modulation after electrical cardioversion failure in patients with persistent atrial fibrillation: the PACIFIC trial: study design. J Cardiovasc Electrophysiol. (2023) 34:270–8. 10.1111/jce.15761 36434797

[25] Bergonti M Spera FR Ferrero TG Nsahlai M Bonomi A Boris W Anterior mitral line in patients with persistent atrial fibrillation and anterior scar: a multicenter matched comparison-the MiLine study. Heart Rhythm. (2023) 20:658–65. 10.1016/j.hrthm.2023.01.009 36640853

[26] Solimene F Compagnucci P Tondo C La Fazia VM Schillaci V Mohanty S Direct epicardial validation of posterior wall electroporation in persistent atrial fibrillation. JACC Clin Electrophysiol. (2024) 10(6):1200–2. 10.1016/j.jacep.2024.04.003 38678453

[27] Della Rocca DG Marcon L Magnocavallo M Menè R Pannone L Mohanty S Pulsed electric field, cryoballoon, and radiofrequency for paroxysmal atrial fibrillation ablation: a propensity score-matched comparison. Europace. (2023) 26(1):euae016. 10.1093/europace/euae016 38245007 PMC10823352

[28] Masuda M Asai M Iida O Okamoto S Ishihara T Nanto K Low-voltage-area ablation in paroxysmal atrial fibrillation: extended follow-up results of the volcano trial. Circ J. (2022) 86:245–52. 10.1253/circj.CJ-21-0476 34321377

[29] Moustafa A Karim S Kahaly O Elzanaty A Meenakshisundaram C Abi-Saleh B Low voltage area guided substrate modification in nonparoxysmal atrial fibrillation: a systematic review and meta-analysis. J Cardiovasc Electrophysiol. (2023) 34:455–64. 10.1111/jce.15764 36453469

[30] Frontera A Pagani S Limite LR Peirone A Fioravanti F Enache B Slow conduction corridors and pivot sites characterize the electrical remodeling in atrial fibrillation. JACC Clin Electrophysiol. (2022) 8:561–77. 10.1016/j.jacep.2022.01.019 35589168

[31] Frontera A Limite LR Pagani S Cireddu M Vlachos K Martin C Electrogram fractionation during sinus rhythm occurs in normal voltage atrial tissue in patients with atrial fibrillation. Pacing Clin Electrophysiol. (2022) 45:219–28. 10.1111/pace.14425 34919281

[32] Hindricks G Potpara T Dagres N Arbelo E Bax JJ Blomström-Lundqvist C 2020 ESC guidelines for the diagnosis and management of atrial fibrillation developed in collaboration with the European association of cardio-thoracic surgery (EACTS). Eur Heart J. (2021) 42:373–498. 10.1093/eurheartj/ehaa612 32860505

