# Supplementary material for: Maintenance of sinus rhythm after electrical cardioversion to identify patients with persistent atrial fibrillation who respond favorably to pulmonary vein isolation: the pre-pacific study
Source: Front Cardiovasc Med. 2024 Oct 11;11:1416975. doi: 10.3389/fcvm.2024.1416975 (PMC11502360; doi:10.3389/fcvm.2024.1416975)
Supplement: Supplementary file 1 [file Datasheet1.docx]

**Supplementary** **Table 1. Univariable and multivariable predictors of ECV working group.**

BSA = body surface area, AAD = anti-arrhythmic drugs, AF= atrial fibrillation, LA=left, LVEF= left ventricular ejection fraction.

|  | Univariable | | | | Multivariable | | | |
| --- | --- | --- | --- | --- | --- | --- | --- | --- |
|  |  |  | 95 CI | |  |  | 95 CI | |
|  | P value | OR | lower | upper | P value | OR | lower | upper |
| **BSA Indexed LA volume** | 0.02 | 0.98 | 0,97 | 0.99 | 0.10 | 0.98 | 0.97 | 1.01 |
| LVEF<40% | 0.30 | 1.50 | 0.69 | 3.26 |  |  |  |  |
| **Age** | 0.09 | 1.03 | 0.99 | 1.07 | 0.23 | 1.03 | 0.98 | 1.01 |
| Female Sex | 0.91 | 1.04 | 0.51 | 2.11 |  |  |  |  |
| Diabetes mellitus | 0.33 | 1.47 | 0.67 | 3.25 |  |  |  |  |
| AAD | 0.98 | 0.99 | 0.48 | 2.04 |  |  |  |  |
| **CHA_2_DS_2_-VASc score** | 0.02 | 1.31 | 1,04 | 1.65 | 0.01 | 1.39 | 1.08 | 1.79 |
| Obstructive sleep apnea | 0.18 | 1.72 | 0.77 | 3.86 |  |  |  |  |
| AF duration | 0.93 | 1.00 | 0.99 | 1.01 |  |  |  |  |
| Previous AF ablation | 0.87 | 1.06 | 0.51 | 2.20 |  |  |  |  |
| **History of tachycardiomyopathy** | 0.09 | 1.86 | 0.91 | 3.81 | 0.37 | 1.43 | 0.65 | 3.15 |
| **Low-voltage areas** | 0.001 | 0.29 | 0.15 | 0.59 | 0.001 | 0.29 | 0.14 | 0.62 |

**Supplementary** **Table 2. Univariable and multivariable predictors of low-voltage area.**

|  | Univariable | | | | Multivariable | | | |
| --- | --- | --- | --- | --- | --- | --- | --- | --- |
|  |  |  | 95 CI | |  |  | 95 CI | |
|  | P value | OR | lower | upper | P value | OR | lower | upper |
| **ECV failure group** | 0.002 | 3.11 | 1.54 | 6.28 | 0.002 | 3.25 | 1.53 | 6.89 |
| LAVI | 0.013 | 1.02 | 1.00 | 1.03 | 0.08 | 1.01 | 0.99 | 1.03 |
| LVEF | 0.98 | 0.99 | 0.97 | 1.03 |  |  |  |  |
| Age | 0.28 | 1.02 | 0.98 | 1.06 |  |  |  |  |
| Female Sex | 0.08 | 1.88 | 0.93 | 3.82 | 0.79 | 1.12 | 0.49 | 2.51 |
| Diabetes mellitus | 0.35 | 1.46 | 0.66 | 3.21 |  |  |  |  |
| Coronary artery disease | 0.98 | 0.99 | 0.48 | 2.04 |  |  |  |  |
| CHA_2_DS_2_-VASc score | 0.29 | 1.13 | 0.90 | 1.41 |  |  |  |  |
| Body mass index | 0.22 | 0.96 | 0.89 | 1.03 |  |  |  |  |
| Obstructive sleep apnea | 0.89 | 1.05 | 0.47 | 2.35 |  |  |  |  |
| AF duration | 0.11 | 1.00 | 0.99 | 1.01 |  |  |  |  |
| Previous AF ablation | 0.05 | 2.14 | 1.01 | 4.51 | 0.08 | 2.10 | 0.93 | 4.73 |

ECV = electrical cardioversion, AF= atrial fibrillation, LAVI=left atrium volum indexed for body surface area, LVEF= left ventricular ejection fraction.

**Supplementary Figure 1.** Kaplan-Meier survival estimates from AF recurrence in the study population stratified by first or REDO procedure.
